# Ovarian cancer survival by residual disease following cytoreductive surgery: a nationwide study in Norway

**DOI:** 10.1038/s41416-025-03018-0

**Published:** 2025-04-26

**Authors:** Cassia B. Trewin-Nybråten, Sigrid Leithe, Torbjørn Paulsen, Hilde Langseth, Renée Turzanski Fortner

**Affiliations:** 1 Department of Registration, Cancer Registry of Norway, Norwegian Institute of Public Health, Oslo, Norway; 2 Department of Gynecological Oncology, Division of Cancer Medicine, Oslo University Hospital, Oslo, Norway; 3 Department of Research, Cancer Registry of Norway, Norwegian Institute of Public Health, Oslo, Norway; 4 Department of Epidemiology and Biostatistics, School of Public Health, Imperial College London, London, UK; 5 Division of Cancer Epidemiology, German Cancer Research Center, Heidelberg, Germany

**Keywords:** Ovarian cancer, Epidemiology

## Abstract

**Background:**

Residual disease (RD) following cytoreductive surgery is prognostic for epithelial ovarian cancer (EOC) patients. Few studies have evaluated RD and survival by tumor histotype and across continuous RD diameter.

**Methods:**

2608 individuals with stages III-IV invasive EOC diagnosed between 2013 and 2022 were identified using the Cancer Registry of Norway. In flexible parametric models, we compared excess mortality comparing RD versus no macroscopic residual disease (NMRD); systemic anti-cancer therapy was evaluated in a sub-cohort from 2019. Excess mortality was assessed across continuous RD size using restricted cubic splines.

**Results:**

Among 1849 patients with cytoreductive surgery, survival was worse for individuals with RD (vs. NMRD), excess hazard ratio (EHR) = 2.62 (95% confidence interval = (2.27–3.01)); no heterogeneity was observed by histotype (*p* = 0.21). Patients with 0.1–0.4 cm RD had 2-fold higher risk of death (EHR = 2.09 (1.63–2.68)) relative to women with NMRD; ~3-fold higher risk was observed for all other categories (e.g., 0.5–0.9 cm, EHR = 2.97 (2.26–3.89); 3.0–20 cm, 2.75 (2.05–3.70)). No significant difference in three-year survival was observed across continuous RD diameter (*p* ≥ 0.17). NMRD was associated with better survival regardless of neoadjuvant chemotherapy.

**Discussion:**

Achieving NMRD resulted in the best survival outcomes. Among patients with RD, we observed no significant difference in survival by RD diameter.

## Introduction

Most invasive epithelial tubo-ovarian or primary peritoneal cancer (collectively referred to as epithelial ovarian cancer; EOC) patients are treated with cytoreductive surgery [1]. Residual disease (RD) following primary (PCS) or interval (ICS) cytoreductive surgery is prognostic, as has been shown previously [2–4]. A limitation of prior studies is a lack of data on survival following surgery by tumor histotype, despite difference in response to treatment, progression, and outcomes by disease subtype [4, 5]. Further, while cutpoints of 0 cm vs. <1 cm or ≥1 cm are commonly used [2], few studies have considered RD by more refined categories [6, 7] or as a continuous measure.

Consistent with contemporary treatment guidelines in many regions, cytoreductive surgery is the first-line EOC treatment in Norway, followed by chemotherapy with a platinum-based agent (predominantly carboplatin) and a taxane (predominantly paclitaxel) [1]. Dependent on patient and tumor characteristics, bevacizumab may be administered with chemotherapy and used in maintenance therapy (patients inoperable, stage IV, stage III RD > 1 cm following primary surgery, or received neoadjuvant chemotherapy), with poly (ADP-ribose) polymerase inhibitors (PARPi) used in maintenance therapy for select patient groups (i.e., platinum-sensitive high-grade EOC in the context of homologous recombination deficiency (HRD) positive or unknown). Neoadjuvant chemotherapy (NACT) is given when there are contraindications to primary surgery or surgery is delayed.

The aims of this study were to evaluate survival among invasive EOC diagnosed at stages III or IV by RD status following cytoreductive surgery and histotype, and across recorded RD diameter. We further evaluated RD and survival in the context of systemic anti-cancer therapy (SACT), including NACT.

## Methods

We used data from the Cancer Registry of Norway (CRN), the Clinical Registry for Gynecological Cancer, and the Central Population Register. The CRN, established in 1951, includes incident cancer diagnoses with mandatory reporting since 1953. The CRN follows international standards for coding and classification and completeness was estimated to be 99.8% for ovarian cancer (C56, C57.0–4, C48.2) and 98.6% for all sites (C00–C96) during 2018–2022 [8]. Tumors (site and morphology) are classified according to ICD-O-3 (International Classification of Diseases for Oncology 3^rd^ edition). The CRN is regularly updated with migration and mortality data from the Central Population Register.

### Study population: Norwegian Clinical Registry for Gynecological Cancer

The Clinical Registry for Gynecological Cancer has been described previously [4]. Briefly, this registry was centralized at the Cancer Registry of Norway in 2012 and for “ovarian” cancer includes all cases of ovarian (C56), fallopian tube (C57), and primary peritoneal (48.2) cancers; these cancers are subsequently referred to as ovarian cancer. The registry includes pathological data on tumor localization, morphology, grade and International Federation of Gynecology and Obstetrics (FIGO) stage at diagnosis. Hospitals submit clinical diagnostic reports including number of co-morbidities and Eastern Cooperative Oncology Group (ECOG) performance status [9] and clinical surgical reports including type of surgery, diameter of RD in the abdominal cavity following cytoreductive surgery, ascites, tumor rupture, and surgical complications. Data on systemic anti-cancer therapies (SACT) administered or prescribed in hospitals, including platinum- and taxane-based chemotherapies, bevacizumab, and PARPi, were available from 2019 for all regions except northern Norway (10% of the population) through the INSPIRE (INcreaSe PharmaceutIcal REporting) project [10].

Individuals eligible for this study were diagnosed with an incident stage III-IV EOC between January 1, 2013 and December 31, 2022 at age <90 years while residing in Norway. Patients diagnosed in 2012 were excluded given a high proportion of missing data on clinical characteristics and surgical outcomes.

We identified 2824 potentially eligible individuals with EOC. We excluded individuals with no clinical diagnostic report (*n* = 126 (4.5%)), or operated patients with no clinical surgical report (*n* = 90 (4.6%)).

After these exclusions, 2608 individuals remained, 1849 who were operated (main population of interest) and a further 759 not operated but included to evaluate comparability between operated and non-operated patients. A secondary analysis evaluated survival in the context of SACT. Given data availability, this analysis was restricted to 725 operated patients diagnosed from January 1, 2019 (excluded *n* = 1044 diagnosed before this date and *n* = 80 patients in northern Norway (data not available)).

We excluded patients with stages I-II disease; of the 1044 surgically treated stage I/II patients eligible for the study, 918 had clinical surgical reports. Of these, 904 had no RD following surgery and just 4 patients had recorded continuous RD.

Based on previous work [4], our study population size had adequate power to detect expected effect sizes for RD for all histotypes combined and for high-grade serous, but limited power for other histotypes.

### Classification of histotype

Tumors were categorized according to the 4^th^ (2014) edition of the World Health Organization (WHO) classification guidelines for female reproductive tumors [11], as previously described [4]. For serous tumors not classified as low-grade (8460/3) or high-grade (8461/3), grade from the pathology reports was used (“high”-grade (grades = 2–4); “low”-grade (grade = 1)). As has been done previously [4, 5], we classified grade 3 or 4 endometrioid (*n* = 11) or adenocarcinoma (*n* = 67) as high-grade serous given potential misclassification of high-grade serous tumors as endometrioid [12].

### Statistical methods

Survival analyses evaluated the prognostic effect of RD status after cytoreductive surgery. Surgically treated patients were thus followed from date of surgery until the earliest event of emigration, all-cause death, or October 31, 2023 (maximum follow-up = 10.5 years; patients with SACT data, maximum follow-up = 4.8 years). We used flexible parametric models [13, 14] to model relative survival, the ratio of observed survival probability among EOC patients to the expected survival probability of women of the same age, calendar year, and residential health trust in the Norwegian population. Patients missing residential health trust (*n* = 9) were assigned the residential health trust of their diagnostic hospital. The observed survival probability of patients is equivalent to overall survival, which was estimated in supplementary analyses using Kaplan–Meier methods.

Flexible parametric models estimated excess hazard; the ratio of observed to expected mortality (inverse of relative survival) and compared groups with excess hazard ratios (EHR) with 95% confidence intervals (CIs). Based on previous work demonstrating similar baseline hazards within the following subgroups [4], we allowed the baseline hazard to vary by two degrees of freedom for (a) high-grade serous or carcinosarcoma; (b) low-grade serous or endometrioid; (c) mucinous or clear cell; and (d) adenocarcinoma (low/unknown grade or other epithelial. All clinical registry covariates described above were considered for inclusion in the models, as well as demographic characteristics (age, year, and residential health trust at diagnosis). Final models included RD, histotype, stage, age and ECOG performance status at diagnosis. Year of diagnosis was not associated with excess hazard after adjustment for residual disease and was therefore excluded. Wald tests were used to consider the statistical significance of variables.

We modeled RD in three ways: dichotomously, categorically, and continuously. The dichotomous and categorical models compared the excess hazard for patients with RD compared to patients with no macroscopic residual disease (NMRD) after surgery. In dichotomous models, the group with RD was expected to be smaller and have larger variance than the group with no RD. In categorical models, RD diameter was categorized to create similarly sized groups. The analysis of the effect of RD on survival in the context of SACT was restricted to individuals who received adjuvant platinum-based chemotherapy and was stratified by NACT. The continuous model only included patients with RD and aimed to identify any threshold of RD diameter that was important for survival probability. Three-year relative survival was predicted over continuous RD, modeled on a log scale by restricted cubic splines with 3 degrees of freedom. The boundary knots were set to 0.2 cm and 2.0 cm due to sparse data beyond these diameters. Survival across continuous RD was modeled for high-grade serous tumors and for all tumors combined (sample size precluded modeling other histotypes even as a composite group).

From dichotomous models, we also predicted histotype-specific relative survival (limited to high- and low-grade serous, carcinosarcoma, mucinous, and clear cell tumors due to sample size) and NACT-specific relative survival for patients with and without RD. For the latter predictions, the baseline hazard varied by two degrees of freedom in each strata of NACT and RD status. All relative survival predictions were made for stage III patients with median age at diagnosis and ECOG = 0 (fully active with no restrictions).

Data were analyzed at the CRN using STATA version 18 [15]. Two-sided *P*-values < 0.05 were considered statistically significant.

## Results

### Baseline characteristics

Of the 2608 eligible patients, 70.9% (*n* = 1849) had cytoreductive surgery. The proportion operated varied by patient characteristics, with highest proportion operated among patients with tumors of mucinous, endometrioid, and carcinosarcoma histology (>84% vs. 72% high-grade serous), stage III disease (78.9% vs. 56.9% stage IV), younger age at diagnosis (90.6% ages 40–49 vs. 66.7% ages 70–79), and low ECOG performance score (Score = 0, 79.1% operated vs. Score = 2, 46.3%; Table 1). Among women with cytoreductive surgery, histotype, stage, year of diagnosis, and ECOG score were all associated with achievement of NMRD, with highest proportion of NMRD among women with endometrioid tumors (84.6%), and those with stage III disease (64.7%), more recent diagnosis (72.4%), and ECOG score = 0 (68.4%). A somewhat higher proportion of those diagnosed with tumor localization as the fallopian tube had NMRD (64.7%), relative to peritoneum (61.2%) or ovary (59.2%). Most patients were diagnosed with stage III disease (70.8%), regardless of histotype (range 69.2–92.9%) (Supplementary Table 1). The majority of patients were diagnosed with tumor localization in the fallopian tube (50.5%), ranging from <5% of clear cell, mucinous, or endometrioid tumors to 57.3% of high-grade serous tumors.

**Table 1:** Surgical and residual disease status by patient characteristics.

Characteristic	Patients, *N*	Cytoreductive surgery, %	Residual disease status, *N* (%)			*P*-value	Diameter of residual disease in abdominal cavity^a^, cm			
			Operated patients^b^	No residual disease	Residual disease		Median	Mean	SD	Maximum
Total	2608	70.9%	1849 (100%)	1154 (62.4%)	643 (34.8%)		1.0	1.6	2.3	>15
Tumor localization						0.15				
Peritoneum (C48.2)	150	68.7%	103 (100%)	63 (61.2%)	37 (35.9%)		0.9	2.5	4.0	>15
Ovary (C56)	1222	64.8%	792 (100%)	469 (59.2%)	294 (37.1%)		1.0	1.7	2.4	>15
Tube (C57.0)	938	97.4%	914 (100%)	591 (64.7%)	304 (33.3%)		1.0	1.3	1.6	11
Unknown (C57.9)	298	13.4%	40 (100%)	31 (77.5%)	8 (20.0%)		1.0	2.3	3.4	10
Histotype						0.02				
High-grade Serous	2112	72.0%	1521 (100%)	944 (62.1%)	543 (35.7%)		1.0	1.6	2.4	>15
Low-grade Serous	161	78.9%	127 (100%)	87 (68.5%)	31 (24.4%)		1.0	1.1	1.3	6
Carcinosarcoma	76	84.2%	64 (100%)	35 (54.7%)	25 (39.1%)		1.0	1.0	0.7	3
Mucinous	32	90.6%	29 (100%)	14 (48.3%)	14 (48.3%)		1.0	2.2	3.0	10
Clear cell	46	82.6%	38 (100%)	21 (55.3%)	15 (39.5%)		2.0	2.1	1.3	4
Endometroid	30	86.7%	26 (100%)	22 (84.6%)	3 (11.5%)					
Adenocarcinoma (low/unknown grade)	117	25.6%	30 (100%)	21 (70.0%)	9 (30.0%)		1.0	2.2	1.8	5
Other epithelial	34	41.2%	14 (100%)	10 (71.4%)	3 (21.4%)					
Stage						<0.01				
III	1660	78.9%	1310 (100%)	848 (64.7%)	428 (32.7%)		1.0	1.6	2.3	>15
IV	948	56.9%	539 (100%)	306 (56.8%)	215 (39.9%)		0.9	1.4	2.3	>15
Age at diagnosis						0.13				
<40	50	94.0%	47 (100%)	32 (68.1%)	13 (27.7%)		1.0	1.1	0.8	2.5
40–49	160	90.6%	145 (100%)	98 (67.6%)	44 (30.3%)		1.0	1.2	1.2	5
50–59	440	83.9%	369 (100%)	229 (62.1%)	132 (35.8%)		1.0	1.7	2.4	>15
60–69	814	76.3%	621 (100%)	393 (63.3%)	210 (33.8%)		1.0	1.8	2.6	>15
70–79	850	66.7%	567 (100%)	351 (61.9%)	198 (34.9%)		1.0	1.3	1.6	10
80–89	294	34.0%	100 (100%)	51 (51.0%)	46 (46.0%)		1.0	1.9	3.5	>15
Year of diagnosis						<0.01				
2013–2015	642	72.3%	464 (100%)	219 (47.2%)	223 (48.1%)		1.0	2.0	3.1	>15
2016–2018	789	73.5%	580 (100%)	352 (60.7%)	211 (36.4%)		1.0	1.4	1.8	11
2019–2022	1177	68.4%	805 (100%)	583 (72.4%)	209 (26.0%)		1.0	1.3	1.7	10
Residential health region						<0.01				
South-East	1469	65.0%	955 (100%)	667 (69.8%)	274 (28.7%)		1.0	1.4	1.9	13
West	555	78.7%	437 (100%)	204 (46.7%)	209 (47.8%)		1.0	1.9	3.0	>15
Mid	311	88.7%	276 (100%)	145 (52.5%)	122 (44.2%)		1.0	1.4	1.2	6
North	273	66.3%	181 (100%)	138 (76.2%)	38 (21.0%)		0.4	1.3	3.2	>15
ECOG performance status						<0.01				
0 (fully active)	1484	79.1%	1174 (100%)	803 (68.4%)	349 (29.7%)		1.0	1.5	1.9	13
1 (restricted activity)	699	70.2%	491 (100%)	251 (51.1%)	218 (44.4%)		1.0	1.6	2.6	>15
2 (unable to work)	160	46.3%	74 (100%)	39 (52.7%)	32 (43.2%)		1.0	1.2	1.1	4
3-4 (Limited self-care or fully disabled)	99	14.1%	14 (100%)	7 (50.0%)	5 (35.7%)		1.0	0.9	0.7	1.5
Unknown	166	57.8%	96 (100%)	54 (56.2%)	39 (40.6%)		1.0	2.4	4.1	>15
Number of co-morbidities						0.64				
None	1269	78.7%	978 (100%)	618 (63.2%)	360 (36.8%)		1.0	1.6	2.4	>15
One	806	64.9%	506 (100%)	329 (65.0%)	177 (35.0%)		1.0	1.4	1.7	10
Two	415	65.5%	259 (100%)	171 (66.0%)	88 (34.0%)		0.6	1.5	2.7	>15
Three or more	98	52.0%	50 (100%)	35 (70.0%)	15 (30.0%)		1.0	0.9	0.6	2
Unknown	20	20.0%	4 (100%)	1 (25.0%)	3 (75.0%)					
Type of chemotherapy^c^, *N* = 1050						0.18				
None	101	22.8%	23 (100%)	19 (82.6%)	4 (17.4%)					
Neoadjuvant only	24	100%	24 (100%)	21 (87.5%)	3 (12.5%)					
Neoadjuvant and adjuvant	313	100%	313 (100%)	216 (69.0%)	91 (29.1%)		1.0	1.4	1.7	10
Adjuvant only	365	100%	365 (100%)	266 (72.9%)	94 (25.8%)		1.0	1.5	1.8	10
Neoadjuvant chemotherapy regimen^b^, *N* = 337						<0.01				
Platinum-based + taxane	210	100%	210 (100%)	134 (63.8%)	71 (33.8%)		0.5	1.3	1.4	6
Platinum-based + taxane + bevacizumab	127	100%	127 (100%)	103 (81.1%)	23 (18.1%)		1.0	1.7	2.3	10
Neoadjuvant cycles^b^, *N* = 337						0.58				
1–3	200	100%	200 (100%)	144 (72.0%)	54 (27.0%)		0.9	1.4	1.8	10
4–6	137	100%	137 (100%)	93 (67.9%)	40 (29.2%)		1.0	1.3	1.4	5

Stage III/IV epithelial ovarian cancer, 2013–2022 (*N* = 2608).

*ECOG* Eastern Cooperative Oncology Group.

^a^Residual disease diameter was not shown if <5 patients had residual disease following cytoreductive surgery.

^b^Represents total number of operated patients, including 52 (2.8%) without known residual disease status. Percentages in subsequent columns do not add up to 100% as the denominator includes these 52 patients.

^c^Chemotherapy available for a subset of patients diagnosed during 2019–2022.

### Outcomes by residual disease status

**Fig. 1:**
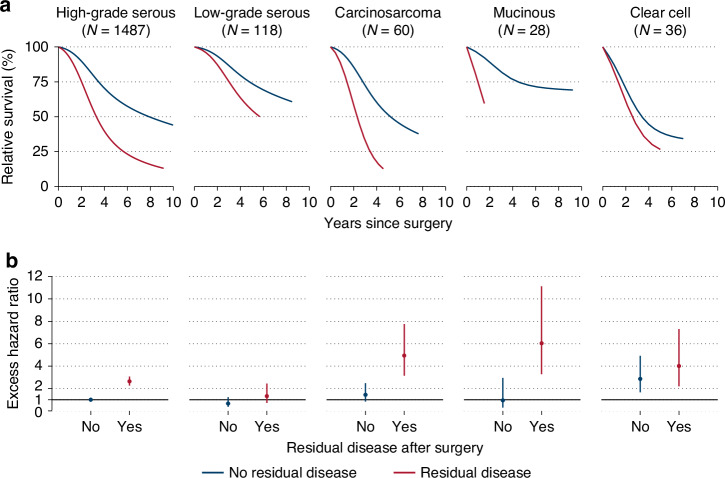
Relative survival by histotype and residual disease status after cytoreductive surgery. **a** Relative survival and **b** excess hazard ratios with 95% confidence intervals, by histotype and residual disease status after debulking surgery. Women diagnosed with stage III/IV epithelial ovarian cancer (EOC) during 2013–2022 (*N* = 1729). Relative survival was predicted from flexible parametric models for women with stage III EOC, median age 64 years and fully active ECOG performance status at diagnosis.

Among patients with cytoreductive surgery, survival outcomes were worse for individuals with RD (relative to NMRD, EHR = 2.62 (2.27–3.01)). Significant associations between RD and survival were observed for high-grade serous (2.64 (2.26–3.07)), carcinosarcoma (3.43 (1.72–6.87)), and mucinous tumors (6.45 (1.79–23.25)), with no statistically significant heterogeneity observed by histotype (*p* = 0.21) (Table 2). Overall, patients with carcinosarcomas, mucinous, or clear cell tumors had worse survival, and low-grade serous and endometrioid better survival, as compared to those with high-grade serous tumors (Supplementary Table 2). Three-year survival ranged from 54.9% (clear cell) to 86.6% (low-grade serous) for patients with NMRD, and from 33.1% (carcinosarcoma) and 43.0% (clear cell) to 75.0% (low-grade serous) for patients with RD (Fig. 1a, Supplementary Table 3). The pattern was similar when Kaplan–Meier estimates were evaluated (Supplementary Fig. 1). We further cross-classified patients by histotype and RD status. Relative to patients with high-grade serous disease and NMRD, RD was more strongly associated with risk of death among mucinous and carcinosarcoma patients (Fig. 1b).

**Table 2:** Excess hazard ratios (EHR) with 95% confidence intervals (CI) for death due to stage III/IV epithelial ovarian cancer according to histotype and residual disease (RD), 2013–2022 (*N* = 1797).

Histotype/Residual disease	Patients/deaths	EHR (95% CI)	*P*-value
Model 1 Adjusted for histotype^a,b^, *N* = 1797			
All histotypes			
No residual disease	1154/470	1 (ref)	
Residual disease	643/481	2.62 (2.27–3.01)	<0.01
Model 2 Stratified by histotype^a,b,c^, *N* = 1729	*Interaction between histotype and residual disease: P* = *0.21*		
High-grade Serous			
No residual disease	944/387	1 (ref)	
Residual disease	543/407	2.64 (2.26–3.07)	<0.01
Low-grade Serous			
No residual disease	87/26	1 (ref)	
Residual disease	31/16	1.99 (0.96–4.13)	0.07
Carcinosarcoma			
No residual disease	35/17	1 (ref)	
Residual disease	25/22	3.43 (1.72–6.87)	<0.01
Mucinous			
No residual disease	14/5	1 (ref)	
Residual disease	14/12	6.45 (1.79–23.25)	<0.01
Clear Cell			
No residual disease	21/16	1 (ref)	
Residual disease	15/13	1.40 (0.64–3.09)	0.40
Model 3 Categorical RD^a,b^, *N* = 1653			
All histotypes			
No residual disease	1154/470	1 (ref)	
0.1–0.4 cm	138/86	2.09 (1.63–2.68)	<0.01
0.5–0.9 cm	89/69	2.97 (2.26–3.89)	<0.01
1.0 cm	102/83	2.74 (2.13–3.53)	<0.01
1.1–2.9 cm	94/61	3.10 (2.32–4.13)	<0.01
3.0–20 cm	78/59	2.75 (2.05–3.70)	<0.01

^a^All estimates were adjusted for age, stage, and Eastern Cooperative Oncology Group (ECOG) performance status.

^b^All models excluded *N* = 52 patients with unknown residual disease status. Model 3 additionally excluded *N* = 144 patients with unknown diameter of residual disease.

^c^Model 2 also excluded *N* = 68 patients with endometrioid, low-grade adenocarcinoma and other epithelial histologies due to too few patients to estimate histotype-specific effects of RD status.

We next evaluated risk of death by approximate quintiles of diameter of RD. Individuals with 0.1–0.4 cm RD had approximately 2-fold higher risk of death (EHR = 2.09 (1.63–2.68)) relative to women with NMRD. Approximately 3-fold higher risk was observed for all other RD groups (e.g., 0.5–0.9 cm, EHR = 2.97 (2.26–3.89); 3.0–20 cm, 2.75 (2.05–3.70)) (Table 2).

**Fig. 2:**
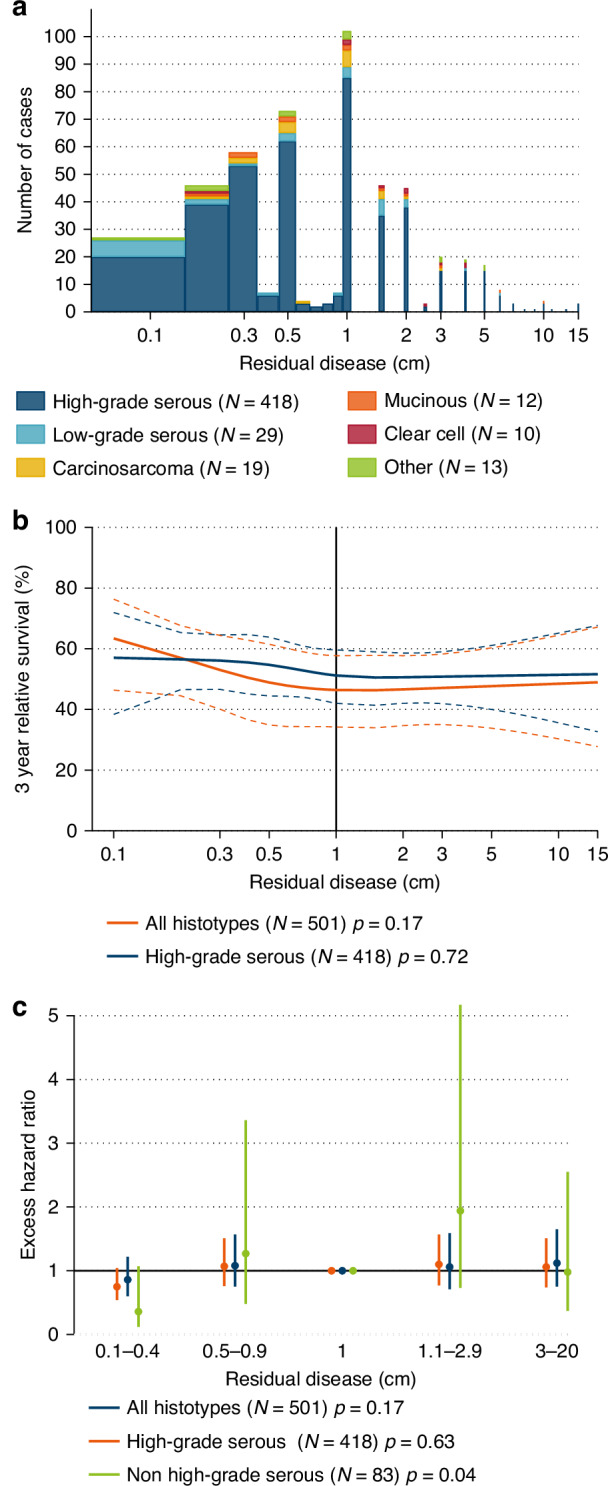
Residual disease diameter and relative survival by histotype and diameter of residual disease after cytoreductive surgery. **a** Residual disease diameter, **b** three-year relative survival, and **c** excess hazard ratios with 95% confidence intervals, by histotype and residual disease diameter. Patients with residual disease following cytoreductive surgery for stage III/IV epithelial ovarian cancer (EOC) during 2013–2022 (*N* = 501). In panel (**b**), three-year relative survival was predicted for women with stage III EOC, median age 64 years and fully active ECOG performance status at diagnosis. Predictions were made from flexible models with the log of residual disease diameter modeled as a restricted cubic spline with three degrees of freedom. The first model included all histotypes and was adjusted for histotype (high-grade serous vs non high-grade serous). Predictions are shown for non high-grade serous histotypes. The second model was restricted to patients with high-grade serous tumors.

RD diameter among those with remaining tumor was median 1.0 cm (Fig. 2a). RD up to 0.5 cm appeared to be reported to the nearest 0.1 cm, whereas RD above 0.5 cm were largely rounded to the nearest half or full cm. Notably, 20% of all patients with RD following cytoreductive surgery were recorded with exactly 1.0 cm. When RD was modeled continuously with a restricted cubic spline, no significant difference in three-year survival was observed across continuous RD diameter for all patients (*p* = 0.17) or for high-grade serous disease (*p* = 0.72) (Fig. 2b; three-year relative survival estimates for 0.2, 0.5, 1.0, and 2.0 cm RD Supplementary Table 4). Finally, EHRs were evaluated for all patients, and subgroups of high-grade serous and other histologies by RD diameter in categories, with exactly 1.0 cm RD as the reference. While none of the individual RD categories were statistically significant (e.g., 0.1–0.4 vs. 1.0 cm, high-grade serous, EHR = 0.86 (0.60–1.22); “other” histologies, EHR = 0.36 (0.12–1.07)), there was an overall significant association (*p* = 0.04) between RD size and survival for the non-high grade serous histotype group (Fig. 2c).

### Sub-cohort with SACT data

#### Sub-cohort baseline characteristics

Among individuals diagnosed after 2019, operated, and with SACT data available (*n* = 725), 96.8% received chemotherapy, with 3.3% receiving neoadjuvant only, 43.2% receiving neoadjuvant and adjuvant, and 50.3% receiving adjuvant only (Supplementary Table 5). Among women who received neoadjuvant chemotherapy (*n* = 337), 37.7% were treated with platinum- and taxane-based chemotherapies together with bevacizumab (22.3% 1-3 cycles; 15.4% 4-6 cycles). The remainder were treated with platinum- and taxane-based chemotherapies alone (62.3%; 37.1% 1-3 cycles, 24.9% 4-6 cycles, 0.3% >6 cycles). In the adjuvant setting, 28.8% of patients were treated with bevacizumab, 13.9% were treated with PARPi, and 10.0% with both. Of women defined as high risk of recurrence based on Norwegian guidelines (neoadjuvant chemotherapy, stage III and >1 cm residual tumor, or stage IV), 39.9% were treated with bevacizumab, 10.8% with PARPi, and 13.8% with both. Conversely, among women not in the high-risk group, 8.7% were treated with bevacizumab, 19.5% with PARPi, or 3.3% with both (data not tabled).

#### Outcomes by Residual Disease Status in the Context of Treatment

**Fig. 3:**
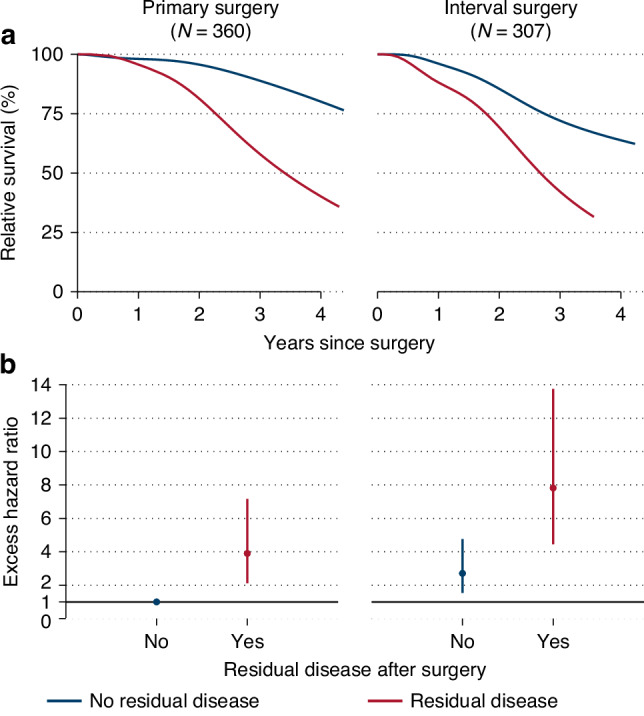
Relative survival by neoadjuvant chemotherapy and residual disease status after cytoreductive surgery. **a** Predicted relative survival and **b** excess hazard ratios with 95% confidence intervals, by residual disease status for (i) patients treated with primary cytoreductive surgery (PCS) followed by chemotherapy or (ii) neadjuvant chemotherapy and interval cytoreductive surgery (ICS). Patients with stage III/IV invasive epithelial ovarian cancer who received adjuvant chemotherapy, 2019–2022 (*N* = 667). Panel (**a**) shows relative survival predicted for patients aged 64 years at diagnosis, stage III high-grade serous histotype and ECOG performance status zero (fully active). Panel (**b**) shows EHR with PCS and no residual disease as the reference. Adjusted for histotype, stage, age, ECOG performance status and adjuvant bevacizumab or PARP-inhibitors.

Among women with PCS (i.e. no NACT), patients with 0.1–0.9 cm diameter RD had highest risk of death (e.g., EHRs: 0.1–0.9 cm, 8.72 (4.59–16.55); 1.1–20 cm, 3.64 (1.87–7.10)) (Table 3). This pattern was not evident among patients with ICS (i.e. received NACT) (e.g., EHRs: 0.1–0.9 cm, 2.09 (1.18–3.71); 1.1–20 cm, 3.00 (1.45–6.19)) (p-interaction=0.01). Associations were similar when mucinous tumors were excluded from the overall EOC group, and when restricted to high-grade serous disease (Table 3). In mutually adjusted models (including diameter of RD), neoadjuvant chemotherapy, carcinosarcoma, mucinous or clear cell histology, stage IV, ECOG > 0, and higher age at diagnosis were associated with poorer survival, whereas PARPi treatment was associated with better survival. Achieving NMRD was associated with better survival, regardless of whether PCS or NACT with ICS (Fig. 3a; one- and three-year survival estimates in Supplementary Table 6); patients with ICS and NMRD had worse outcomes than those with PCS and NMRD (Fig. 3b). The pattern was similar when Kaplan–Meier estimates were evaluated (Supplementary Fig. 2**)**.

**Table 3:** Excess hazard ratios (EHR) with 95% confidence intervals (CI) for death due to stage III/IV epithelial ovarian cancer in the context of systemic anti-cancer therapy.

Variable	All histotypes^a^, *N* = 645			High-grade serous^b^, *N* = 550		
	Patients/ deaths	Adjusted EHR^b^ (95% CI)	*P*-value	Patients/ deaths	Adjusted EHR^b^ (95% CI)	*P*-value
Neoadjuvant chemotherapy and residual disease						
Primary surgery and adjuvant chemotherapy						
No residual disease	266/44	1 (ref)	<0.01	211/33	1 (ref)	<0.01
0.1–0.9 cm	33/18	8.72 (4.59–16.55)		28/16	9.38 (4.58–19.23)	
1.0 cm	14/6	1.99 (0.73–5.46)		10/4	2.06 (0.57–7.40)	
1.1–20.0 cm	37/15	3.64 (1.87–7.10)		28/9	3.29 (1.41–7.69)	
Neoadjuvant chemotherapy and interval cytoreductive surgery						
No residual disease	216/61	1 (ref)		200/55	1 (ref)	
0.1–0.9 cm	38/18	2.09 (1.18–3.71)		35/16	1.99 (1.09–3.66)	
1.0 cm	13/10	3.81 (1.83–7.92)		12/9	3.71 (1.73–7.93)	
1.1–20.0 cm	28/12	3.00 (1.45–6.19)		26/11	3.48 (1.71–7.08)	
Neoadjuvant chemotherapy						
No	350/83	1 (ref)	<0.01	277/62	1 (ref)	<0.01
Yes	295/101	2.64 (1.65–4.25)		273/91	2.78 (1.62–4.78)	
Adjuvant Bevacizumab or PARP-inhibitor						
Neither	306/79	1 (ref)	0.01	242/61	1 (ref)	0.06
Bevacizumab	186/53	0.74 (0.49–1.11)		161/42	0.75 (0.47–1.19)	
PARP-inhibitor	91/25	0.42 (0.25–0.71)		87/24	0.46 (0.26–0.81)	
Both	62/27	0.59 (0.35–1.01)		60/26	0.68 (0.39–1.19)	
Stage						
III	425/108	1 (ref)	0.03	355/87	1 (ref)	0.05
IV	220/76	1.45 (1.04–2.04)		195/66	1.46 (1.01–2.11)	
ECOG performance status						
0 (fully active)	456/117	1 (ref)	0.04	382/93	1 (ref)	0.04
1 (restricted activity)	127/49	1.64 (1.13–2.39)		118/45	1.74 (1.17–2.58)	
2–4 (unable to work – fully disabled)	35/13	1.84 (0.96–3.54)		29/10	1.61 (0.76–3.44)	
Unknown	27/5	1.35 (0.48–3.82)		21/5	1.75 (0.63–4.84)	
Age at diagnosis (per year)	645/184	1.01 (1.00–1.03)	0.15	550/153	1.01 (1.00–1.03)	0.13
Histotype						
High-grade Serous	550/153	1 (ref)	<0.01			
Low-grade Serous	34/3	0.51 (0.14–1.83)				
Carcinosarcoma	22/11	3.29 (1.67–6.51)				
Mucinous	9/6	6.30 (2.60–15.24)				
Clear cell	6/5	6.34 (2.28–17.64)				
Endometroid	7/0	N/A				
Adenocarcinoma (low/unknown grade)	14/6	3.09 (1.22–7.84)				
Other epithelial	3/0	N/A				

Patients who received adjuvant chemotherapy after cytoreductive surgery, 2019–2022 (*N* = 645).

*PARP* Poly ADP Ribose Polymerase, *ECOG* Eastern Cooperative Oncology Group.

^a^The model including all histotypes excluded *N* = 80 patients residing in Northern Norway (no chemotherapy data).; *N* = 47 patients who did not receive adjuvant chemotherapy and *N* = 33 patients with unknown residual disease diameter. The baseline hazard deviated by two degrees of freedom for high-grade serous versus other histotypes. Excess hazard ratios were adjusted for all factors shown in the table.

^b^The high-grade serous model excluded *N* = 64 patients residing in Northern Norway (no chemotherapy data).; *N* = 37 patients who did not receive adjuvant chemotherapy and *N* = 29 patients with unknown residual disease diameter. Excess hazard ratios were adjusted for all factors shown in the table.

## Discussion

RD was associated with poorer EOC survival overall, with associations observed across histotypes. Worse survival was observed with reported RD overall (i.e., regardless of size), and also with RD below the threshold of “optimal” cytoreduction (i.e., <1 cm, as compared to NMRD). These findings are in line with a recent meta-analysis, which observed best survival with NMRD and no clear associations with other thresholds of RD diameter, though limited studies with data beyond the standard thresholds were included [16]. In the current study, the overall pattern of worse survival with any diameter of RD was observed when further adjusting for SACT and when stratifying by administration of NACT. A different pattern of associations was observed between size of RD < 1 or ≥1 cm and survival in patient subgroups defined by NACT (i.e., with PCS or ICS).

Somewhat stronger associations were observed between RD and survival for patients with mucinous and carcinosarcoma histologies, though analyses by histotype were limited by sample size. Outcomes following surgery are a function of patterns of disease dissemination and RD burden together with underlying tumor biology, response to treatment, and individual patient characteristics. These findings are partly in line with a prior study by histotype (stages IIIC and IV); the prior study observed a larger difference between NMRD and RD for mucinous and clear cell, as compared to serous tumors, though the differences by histology in that study were not statistically significant [17]. RD, as compared to NMRD, was associated with worse survival. We observed no differences in survival across reported RD diameter among women with RD > 0 cm in the current study; results were consistent when restricted to patients with high-grade serous tumors.

We observed no significant associations by RD diameter for the other histotypes (evaluated as a group due to sample size), though with suggestively better survival among patients with smaller reported RD (≤0.4 cm) in this subgroup. Our observation of a potentially stronger impact of remaining RD on survival for mucinous tumors and carcinosarcomas is in line with these subtypes’ limited response to chemotherapy and the few options for targeted therapies [18, 19]. In contrast, contemporary treatments such as PARPi have some efficacy for treatment in particular in subsets of high-grade serous tumors [20], with further limited data suggesting potentially stronger effect of bevacizumab for serous as compared to non-serous subtypes [21]. This study adds to the limited data on survival by surgical cytoreduction status for select disease histotypes, and demonstrates the impact of RD on survival outcomes in a population-based setting. Further studies with better representation of histotypes beyond high-grade serous are needed.

NMRD was associated with the longest survival in both the subgroups of patients with PCS and those with ICS following NACT, in analyses restricted to the population with data on SACT (patients diagnosed in or after 2019). When considering amount of RD < 1 or ≥1 cm by NACT status, we observed a different pattern of associations in patients who received NACT and in those who did not. Bevacizumab was indicated for use in Norway in “high risk” patients (stage III and >1 cm RD, stage IV, or neoadjuvant chemotherapy) in the full period considered in this analysis. Strikingly, we observed highest risk of death among patients with RD of 0.1–0.9 cm and who had not received NACT (i.e., outside the “high-risk” subgroup for whom bevacizumab would be indicated). This observation is in line with discussions around definitions of a “low risk” population of patients with advanced stage EOC [22], in light of the high risk of recurrence for all patients diagnosed with advanced disease. While these analyses were based on small numbers (*n* = 465), these findings from an unselected population-based registry study add to the clinical trial data on bevacizumab [23]. PARPi was in use in Norway during the period included in this study, for high-grade tumors sensitive to platinum-based chemotherapy and in the context of a BRCA mutation or HRD-positivity. PARPi use was associated with significantly better survival for high-grade serous tumors, and EOC overall, though only five patients in the overall EOC group had non-high-grade serous histology.

This study focused on the 71% of patients treated with cytoreductive surgery in Norway. These findings may not fully generalize to populations with different referral patterns to surgery or with a substantial proportion of women operated outside of specialist centers. Ovarian cancer cytoreductive surgeries in Norway have been conducted at specialist centers since 2005 [24], in line with data showing better outcomes for patients with cytoreductive surgery at higher volume centers [25]. We extend our prior work, which evaluated RD < 1 vs. ≥1 cm [4], to consider RD across the reported spectrum of diameter. These data provide insights into the relative importance of varying amounts of RD. Notably, we observed a propensity toward rounding, with most reported RD measures at whole or half-centimeter diameters. While this may have introduced measurement error, this is unlikely to explain the study findings. Tumors of 1.0 cm were included in a separate category, given the large proportion recorded at this size. Further, the analysis relied on reported residual disease within the abdominal cavity, which may not represent the full burden of residual disease, and the accuracy of reported residual disease may vary by clinician and case. NACT data were only available from 2019, as part of a national program to collect these data for hospital-based NACT [10]. These analyses were limited by sample size; however, this study provides data on surgical outcomes and survival considering NACT use in the “real-world data” setting in an unselected population.

RD following surgery may have differential importance by tumor histology due to a constellation of factors, ranging from patterns of tumor dissemination to tumor biology and chemo-sensitivity. Achieving NMRD resulted in the best survival outcomes across all evaluated subgroups. Among the predominant high-grade serous tumors, amount of RD after surgery did not appear to have a strong impact on survival when SACT was not considered. In the context of SACT, and accounting for other prognostic factors, patients who had not received NACT and with smaller diameter RD (i.e., not “high-risk” and not recommended for bevacizumab) had poor survival. Further population-based data are warranted to inform (re-)consideration of “optimal” cytoreduction and definitions of low- and high-risk populations.

## Supplementary information

Supplementary Tables 1-6

Supplementary Figure 1

Supplementary Figure 2

## Data Availability

Data used in this study are available by application to helsedata.no.

## Code availability

The code used to generate the results that are central to the paper’s conclusions can be accessed at https://github.com/CBTrewin-Nybraten/ovarian-cancer-survival-by-residual-disease.
