# Supplementary material for: Ovarian cancer survival by residual disease following cytoreductive surgery: a nationwide study in Norway
Source: Br J Cancer. 2025 Apr 26;132(12):1158–66. doi: 10.1038/s41416-025-03018-0 (PMC12152144; doi:10.1038/s41416-025-03018-0)
Supplement: Supplementary file 1 — Supplementary Tables 1-6 [file 41416_2025_3018_MOESM1_ESM.docx]

**Supplemental Table 1.** Patient, tumor, and surgical treatment characteristics by histotype. Surgically treated patients with stage III/IV epithelial ovarian cancer, 2013–2022 (N = 1849).

| **Variable** | **Total** | **High-grade serous** | **Low-grade serous** | **Carcino-sarcoma** | **Mucinous** | **Clear Cell** | **Endometrioid** | **Adeno-carcinoma (unknown/ low grade)** | **Other epithelial** |
| --- | --- | --- | --- | --- | --- | --- | --- | --- | --- |
|  | **N (%)** | **N (%)** | **N (%)** | **N (%)** | **N (%)** | **N (%)** | **N (%)** | **N (%)** | **N (%)** |
| **Total** | 1849 (100) | 1521 (100) | 127 (100) | 64 (100) | 29 (100) | 38 (100) | 26 (100) | 30 (100) | 14 (100) |
| **Age at diagnosis, mean (SD)** | 64 (11) | 64 (11) | 58 (15) | 67 (8) | 59 (12) | 61 (14) | 58 (11) | 61 (12) | 66 (11) |
| **Residual disease in abdominal cavity** |  |  |  |  |  |  |  |  |  |
| No | 1154 (64.2) | 944 (63.5) | 87 (73.7) | 35 (58.3) | 14 (50.0) | 21 (58.3) | 22 (88.0) | 21 (70.0) | 10 (76.9) |
| Yes | 643 (35.8) | 543 (36.5) | 31 (26.3) | 25 (41.7) | 14 (50.0) | 15 (41.7) | 3 (12.0) | 9 (30.0) | 3 (23.1) |
| Unknown | 52 | 34 | 9 | 4 | 1 | 2 | 1 | 0 | 1 |
| **Residual disease diameter^1^ (N = 501)** |  |  |  |  |  |  |  |  |  |
| 0.1–0.4 cm | 138 (27.5) | 118 (28.2) | 10 (34.5) | 3 (15.8) | 3 (25.0) | 1 (10.0) |  | 1 (14.3) |  |
| 0.5–0.9 cm | 89 (17.8) | 76 (18.2) | 4 (13.8) | 5 (26.3) | 2 (16.7) | 0 (0.0) |  | 0 (0.0) |  |
| 1.0 cm | 102 (20.4) | 85 (20.3) | 4 (13.8) | 6 (31.6) | 2 (16.7) | 2 (20.0) |  | 3 (42.9) |  |
| 1.1–2.9 cm | 94 (18.8) | 75 (17.9) | 9 (31.0) | 4 (21.1) | 2 (16.7) | 4 (40.0) |  | 0 (0.0) |  |
| 3–20 cm | 78 (15.6) | 64 (15.3) | 2 (6.9) | 1 (5.3) | 3 (25.0) | 3 (30.0) |  | 3 (42.9) |  |
| Unknown diameter | 142 | 125 | 2 | 6 | 2 | 5 |  | 2 |  |
| **Tumor localization** |  |  |  |  |  |  |  |  |  |
| Peritoneum (C48.2) | 103 (5.7) | 80 (5.4) | 22 (18.0) | 0 (0.0) | 0 (0.0) | 1 (2.6) | 0 (0.0) | 0 (0.0) | 0 (0.0) |
| Ovary (C56) | 792 (43.8) | 557 (37.3) | 81 (66.4) | 35 (54.7) | 29 (100.0) | 36 (94.7) | 25 (96.2) | 18 (72.0) | 11 (84.6) |
| Tube (C57.0) | 914 (50.5) | 855 (57.3) | 19 (15.6) | 29 (45.3) | 0 (0.0) | 1 (2.6) | 1 (3.8) | 7 (28.0) | 2 (15.4) |
| Unknown topography (C57.9) | 40 | 29 | 5 | 0 | 0 | 0 | 0 | 5 | 1 |
| **Stage** |  |  |  |  |  |  |  |  |  |
| III | 1310 (70.8) | 1052 (69.2) | 104 (81.9) | 47 (73.4) | 21 (72.4) | 30 (78.9) | 21 (80.8) | 22 (73.3) | 13 (92.9) |
| IV | 539 (29.2) | 469 (30.8) | 23 (18.1) | 17 (26.6) | 8 (27.6) | 8 (21.1) | 5 (19.2) | 8 (26.7) | 1 (7.1) |
| **Diagnosis period** |  |  |  |  |  |  |  |  |  |
| 2012–2015 | 464 (25.1) | 373 (24.5) | 37 (29.1) | 14 (21.9) | 8 (27.6) | 11 (28.9) | 9 (34.6) | 7 (23.3) | 5 (35.7) |
| 2016–2018 | 580 (31.4) | 468 (30.8) | 39 (30.7) | 25 (39.1) | 8 (27.6) | 17 (44.7) | 9 (34.6) | 9 (30.0) | 5 (35.7) |
| 2019–2022 | 805 (43.5) | 680 (44.7) | 51 (40.2) | 25 (39.1) | 13 (44.8) | 10 (26.3) | 8 (30.8) | 14 (46.7) | 4 (28.6) |
| **Residential health region** |  |  |  |  |  |  |  |  |  |
| South-East | 955 (51.6) | 766 (50.4) | 74 (58.3) | 43 (67.2) | 13 (44.8) | 21 (55.3) | 16 (61.5) | 18 (60.0) | 4 (28.6) |
| West | 437 (23.6) | 369 (24.3) | 23 (18.1) | 9 (14.1) | 10 (34.5) | 10 (26.3) | 3 (11.5) | 7 (23.3) | 6 (42.9) |
| Mid | 276 (14.9) | 238 (15.6) | 20 (15.7) | 9 (14.1) | 1 (3.4) | 2 (5.3) | 3 (11.5) | 2 (6.7) | 1 (7.1) |
| North | 181 (9.8) | 148 (9.7) | 10 (7.9) | 3 (4.7) | 5 (17.2) | 5 (13.2) | 4 (15.4) | 3 (10.0) | 3 (21.4) |
| **ECOG performance status** |  |  |  |  |  |  |  |  |  |
| 0 (fully active) | 1174 (67.0) | 947 (65.8) | 96 (77.4) | 45 (72.6) | 17 (60.7) | 25 (67.6) | 18 (78.3) | 18 (64.3) | 8 (66.7) |
| 1 (restricted activity) | 491 (28.0) | 422 (29.3) | 19 (15.3) | 12 (19.4) | 11 (39.3) | 9 (24.3) | 5 (21.7) | 9 (32.1) | 4 (33.3) |
| 2 (unable to work) | 74 (4.2) | 62 (4.3) | 7 (5.6) | 3 (4.8) | 0 (0.0) | 2 (5.4) | 0 (0.0) | 0 (0.0) | 0 (0.0) |
| 3-4 (Limited self-care or fully disabled) | 14 (0.8) | 8 (0.6) | 2 (1.6) | 2 (3.2) | 0 (0.0) | 1 (2.7) | 0 (0.0) | 1 (3.6) | 0 (0.0) |
| Unknown | 96 | 82 | 3 | 2 | 1 | 1 | 3 | 2 | 2 |
| **Number of co-morbidities** |  |  |  |  |  |  |  |  |  |
| None | 999 (54.1) | 819 (53.9) | 75 (59.5) | 27 (42.2) | 20 (71.4) | 24 (63.2) | 12 (46.2) | 14 (46.7) | 8 (57.1) |
| One | 523 (28.3) | 429 (28.2) | 33 (26.2) | 22 (34.4) | 5 (17.9) | 9 (23.7) | 10 (38.5) | 9 (30.0) | 6 (42.9) |
| Two | 272 (14.7) | 227 (14.9) | 17 (13.5) | 11 (17.2) | 2 (7.1) | 5 (13.2) | 3 (11.5) | 7 (23.3) | 0 (0.0) |
| Three or more | 51 (2.8) | 44 (2.9) | 1 (0.8) | 4 (6.2) | 1 (3.6) | 0 (0.0) | 1 (3.8) | 0 (0.0) | 0 (0.0) |
| Unknown | 4 | 2 | 1 | 0 | 1 | 0 | 0 | 0 | 0 |
| **Ascites** |  |  |  |  |  |  |  |  |  |
| Yes | 822 (48.8) | 703 (50.9) | 53 (46.9) | 18 (30.5) | 7 (25.9) | 12 (32.4) | 10 (40.0) | 15 (53.6) | 4 (30.8) |
| No | 861 (51.2) | 678 (49.1) | 60 (53.1) | 41 (69.5) | 20 (74.1) | 25 (67.6) | 15 (60.0) | 13 (46.4) | 9 (69.2) |
| Unknown | 159 | 135 | 13 | 5 | 1 | 1 | 1 | 2 | 1 |
| **Tumor rupture** |  |  |  |  |  |  |  |  |  |
| No | 1665 (92.5) | 1383 (93.3) | 114 (93.4) | 56 (90.3) | 22 (81.5) | 31 (81.6) | 20 (76.9) | 26 (89.7) | 13 (92.9) |
| Yes | 53 (2.9) | 32 (2.2) | 4 (3.3) | 4 (6.5) | 3 (11.1) | 4 (10.5) | 4 (15.4) | 2 (6.9) | 0 (0.0) |
| Not applicable (advanced disease) | 82 (4.6) | 67 (4.5) | 4 (3.3) | 2 (3.2) | 2 (7.4) | 3 (7.9) | 2 (7.7) | 1 (3.4) | 1 (7.1) |
| Unknown | 42 | 34 | 4 | 2 | 1 | 0 | 0 | 1 | 0 |
| **Perioperative complications** |  |  |  |  |  |  |  |  |  |
| Yes | 1744 (94.7) | 1438 (94.9) | 122 (96.8) | 60 (93.8) | 26 (92.9) | 35 (92.1) | 24 (92.3) | 28 (93.3) | 11 (78.6) |
| No | 97 (5.3) | 77 (5.1) | 4 (3.2) | 4 (6.2) | 2 (7.1) | 3 (7.9) | 2 (7.7) | 2 (6.7) | 3 (21.4) |
| Unknown | 1 | 1 | 0 | 0 | 0 | 0 | 0 | 0 | 0 |
| **Post-operative complications** |  |  |  |  |  |  |  |  |  |
| Yes | 1656 (90.8) | 1370 (91.3) | 106 (84.8) | 56 (90.3) | 26 (92.9) | 35 (92.1) | 24 (92.3) | 26 (89.7) | 13 (92.9) |
| No | 167 (9.2) | 131 (8.7) | 19 (15.2) | 6 (9.7) | 2 (7.1) | 3 (7.9) | 2 (7.7) | 3 (10.3) | 1 (7.1) |
| Unknown | 19 | 15 | 1 | 2 | 0 | 0 | 0 | 1 | 0 |
| **Planned adjuvant chemotherapy** |  |  |  |  |  |  |  |  |  |
| No | 151 (8.3) | 114 (7.6) | 15 (12.1) | 6 (9.4) | 3 (11.5) | 3 (7.9) | 1 (3.8) | 7 (23.3) | 2 (15.4) |
| Yes | 1679 (91.7) | 1395 (92.4) | 109 (87.9) | 58 (90.6) | 23 (88.5) | 35 (92.1) | 25 (96.2) | 23 (76.7) | 11 (84.6) |
| Unknown | 12 | 7 | 2 | 0 | 2 | 0 | 0 | 0 | 1 |

Abbreviations: ECOG: Eastern Cooperative Oncology Group.

^1^Distribution of patients was not shown for histotypes with fewer than 5 patients with known residual disease diameter.

**Supplemental Table 2.** Crude, singularly adjusted and multivariate adjusted excess hazard ratios (EHR) with 95 % confidence intervals (CI) for covariates considered for inclusion in the adjusted residual disease model shown in table 2 (N = 1797).

| **Variable** | **Patients/ deaths** | **Crude estimates** | | **Singularly adjusted for residual disease** | | **Fully adjusted model (model 1 in table 2)** | |  |
| --- | --- | --- | --- | --- | --- | --- | --- | --- |
|  |  |  |  |  |  |  |  |  |
|  |  | **EHR (95% CI)^1^** | **Wald P** | **EHR (95% CI)^1^** | **Wald P** | **EHR (95% CI)^1^** | **Wald P** |  |
| **Residual disease in abdominal cavity** |  |  |  |  |  |  |  |  |
| No residual disease | 1154 / 470 | 1 (ref) | < 0.01 |  |  | 1 (ref) | < 0.01 |  |
| Residual disease | 643 / 481 | 2.82 (2.45–3.25) |  |  |  | 2.62 (2.27–3.01) |  |  |
| **Stage** |  |  |  |  |  |  |  |  |
| III | 1276 / 655 | 1 (ref) | < 0.01 | 1 (ref) | < 0.01 | 1 (ref) | < 0.01 |  |
| IV | 521 / 296 | 1.67 (1.44–1.94) |  | 1.56 (1.34–1.81) |  | 1.57 (1.35–1.83) |  |  |
| **Histotype** |  |  |  |  |  |  |  |  |
| High-grade Serous | 1487 / 794 | 1 (ref) | < 0.01 | 1 (ref) | < 0.01 | 1 (ref) | < 0.01 |  |
| Low-grade Serous | 118 / 42 | 0.41 (0.25–0.69) |  | 0.43 (0.25–0.72) |  | 0.51 (0.30–0.85) |  |  |
| Carcinosarcoma | 60 / 39 | 1.53 (1.08–2.17) |  | 1.57 (1.11–2.22) |  | 1.67 (1.18–2.36) |  |  |
| Mucinous | 28 / 17 | 1.70 (1.00–2.89) |  | 1.67 (0.99–2.83) |  | 1.72 (1.01–2.92) |  |  |
| Clear Cell | 36 / 29 | 2.14 (1.43–3.20) |  | 1.98 (1.32–2.96) |  | 2.03 (1.35–3.04) |  |  |
| Endometroid | 25 / 6 | 0.21 (0.08–0.58) |  | 0.28 (0.11–0.76) |  | 0.32 (0.12–0.84) |  |  |
| Adenocarcinoma (low/unknown grade) | 30 / 18 | 1.28 (0.71–2.28) |  | 1.24 (0.68–2.25) |  | 1.26 (0.70–2.28) |  |  |
| Other epithelial | 13 / 6 | 0.69 (0.26–1.82) |  | 0.76 (0.28–2.04) |  | 0.84 (0.32–2.24) |  |  |
| **ECOG performance status** |  |  |  |  |  |  |  |  |
| 0 (fully active) | 1152 / 537 | 1 (ref) | < 0.01 | 1 (ref) | < 0.01 | 1 (ref) | < 0.01 |  |
| 1 (restricted activity) | 469 / 305 | 1.63 (1.40–1.90) |  | 1.45 (1.24–1.69) |  | 1.42 (1.21–1.65) |  |  |
| 2 (unable to work) | 71 / 39 | 1.85 (1.28–2.68) |  | 1.65 (1.15–2.39) |  | 1.46 (1.01–2.12) |  |  |
| 3-4 (Limited self-care or fully disabled) | 12 / 9 | 3.60 (1.80–7.22) |  | 4.27 (2.15–8.51) |  | 4.40 (2.21–8.74) |  |  |
| Unknown | 93 / 61 | 1.50 (1.12–1.99) |  | 1.34 (1.01–1.79) |  | 1.37 (1.03–1.82) |  |  |
| **Age at diagnosis (per year)** | 1797 / 951 | 1.01 (1.00–1.02) | < 0.01 | 1.01 (1.00–1.02) | < 0.01 | 1.01 (1.00–1.02) | < 0.01 |  |
| **Age group (years)** |  |  |  |  |  |  |  |  |
| < 40 | 45 / 17 | 1 (ref) | 0.12 | 1 (ref) | 0.19 |  |  |  |
| 40–49 | 142 / 62 | 1.28 (0.74–2.20) |  | 1.26 (0.73–2.17) |  |  |  |  |
| 50–59 | 361 / 177 | 1.42 (0.86–2.34) |  | 1.31 (0.79–2.17) |  |  |  |  |
| 60–69 | 603 / 332 | 1.61 (0.98–2.64) |  | 1.51 (0.92–2.47) |  |  |  |  |
| 70–79 | 549 / 302 | 1.61 (0.98–2.65) |  | 1.54 (0.93–2.53) |  |  |  |  |
| 80–89 | 97 / 61 | 1.94 (1.06–3.56) |  | 1.75 (0.96–3.18) |  |  |  |  |
| **Year of diagnosis (per year)** | 1797 / 951 | 0.96 (0.93–0.99) | 0.01 | 1.00 (0.97–1.03) | 0.97 |  |  |  |
| **Diagnosis period** |  |  |  |  |  |  |  |  |
| 2012–2015 | 442 / 350 | 1 (ref) | 0.04 | 1 (ref) | 0.66 |  |  |  |
| 2016–2018 | 563 / 372 | 0.93 (0.79–1.09) |  | 1.07 (0.91–1.26) |  |  |  |  |
| 2019–2022 | 792 / 229 | 0.78 (0.64–0.95) |  | 1.00 (0.82–1.22) |  |  |  |  |
| **Residential health region** |  |  |  |  |  |  |  |  |
| South-East | 941 / 464 | 1 (ref) | < 0.01 | 1 (ref) | 0.37 |  |  |  |
| West | 413 / 243 | 1.38 (1.16–1.63) |  | 1.13 (0.95–1.34) |  |  |  |  |
| Mid | 267 / 158 | 1.28 (1.05–1.56) |  | 1.16 (0.95–1.42) |  |  |  |  |
| North | 176 / 86 | 0.92 (0.71–1.18) |  | 1.05 (0.81–1.36) |  |  |  |  |
| **Tumor location** |  |  |  |  |  |  |  |  |
| Peritoneum (C48.2) | 100 / 56 | 1 (ref) | 0.81 | 1 (ref) | 0.77 |  |  |  |
| Ovary (C56) | 763 / 436 | 1.13 (0.83–1.55) |  | 1.17 (0.86–1.60) |  |  |  |  |
| Tube (C57.0) | 895 / 449 | 1.11 (0.81–1.52) |  | 1.18 (0.87–1.61) |  |  |  |  |
| Unknown topography (C57.9) | 39 / 10 | 0.91 (0.42–1.97) |  | 1.15 (0.53–2.50) |  |  |  |  |
| **Number of co-morbidities** |  |  |  |  |  |  |  |  |
| None | 978 / 523 | 1 (ref) | 0.23 | 1 (ref) | 0.15 |  |  |  |
| One | 506 / 274 | 1.12 (0.95–1.31) |  | 1.19 (1.02–1.40) |  |  |  |  |
| Two | 259 / 128 | 1.02 (0.82–1.27) |  | 1.04 (0.83–1.29) |  |  |  |  |
| Three or more | 50 / 22 | 1.02 (0.61–1.71) |  | 1.18 (0.71–1.96) |  |  |  |  |
| Unknown | 4 / 4 | 2.76 (1.02–7.50) |  | 2.12 (0.78–5.75) |  |  |  |  |
| **Ascites** |  |  |  |  |  |  |  |  |
| No | 804 / 360 | 1 (ref) | < 0.01 | 1 (ref) | 0.79 |  |  |  |
| Yes | 842 / 515 | 1.29 (1.11–1.50) |  | 0.98 (0.84–1.15) |  |  |  |  |
| Unknown | 151 / 76 | 1.39 (1.06–1.83) |  | 1.08 (0.82–1.42) |  |  |  |  |
| **Tumor Rupture** |  |  |  |  |  |  |  |  |
| No | 1627 / 867 | 1 (ref) | 0.13 | 1 (ref) | 0.19 |  |  |  |
| Yes | 52 / 30 | 0.92 (0.61–1.38) |  | 0.91 (0.60–1.36) |  |  |  |  |
| Not applicable (advanced disease) | 80 / 24 | 1.45 (0.93–2.25) |  | 1.53 (0.99–2.36) |  |  |  |  |
| Unknown | 38 / 30 | 1.39 (0.95–2.05) |  | 1.21 (0.82–1.78) |  |  |  |  |
| **Perioperative complication** |  |  |  |  |  |  |  |  |
| No | 1701 / 889 | 1 (ref) | 0.35 | 1 (ref) | 0.48 |  |  |  |
| Yes | 95 / 62 | 1.23 (0.93–1.64) |  | 1.19 (0.90–1.58) |  |  |  |  |
| Unknown | 1 / 0 |  |  |  |  |  |  |  |
| **Post-operative complication** |  |  |  |  |  |  |  |  |
| No | 1616 / 860 | 1 (ref) | 0.71 | 1 (ref) | 0.75 |  |  |  |
| Yes | 163 / 84 | 1.00 (0.78–1.28) |  | 1.00 (0.77–1.28) |  |  |  |  |
| Unknown | 18 / 7 | 0.67 (0.26–1.73) |  | 0.70 (0.28–1.74) |  |  |  |  |

Abbreviations: ECOG: Eastern Cooperative Oncology Group.

^1^N = 52 patients were excluded due to unknown residual disease status after surgery. The baseline hazard deviated by two degrees of freedom for the following subgroups: stage III/IV of (1) High-grade serous/Carcinosarcoma; (2) Low-grade serous/Endometrioid; (3) Mucinous/Clear Cell; (4) Other histologies.

**Supplemental Table 3.** Predicted^1^ relative survival (RS) with 95% confidence intervals (CI) from flexible parametric models, by histotype and residual disease status. Patients diagnosed with stage III/IV invasive epithelial ovarian cancer during 2013-2022 (N = 1729).

| **Histotype/Group** | **Residual disease** | **Patients/ deaths** | **1-year RS (95% CI)** | **3-year RS**^1^ **(95% CI)** | **5-year RS**^1^ **(95% CI)** | **7-year RS**^1^ **(95% CI)** |
| --- | --- | --- | --- | --- | --- | --- |
| **All histotypes** | **No** | **1101 / 116** | **96.8% (96.0–97.4%)** | **79.6% (76.9–82.1%)** | **62.6% (58.7–66.3%)** | **52.6% (48.0–57.0%)** |
|  | **Yes** | **628 / 118** | **91.7% (89.9–93.2%)** | **54.9% (50.1–59.4%)** | **29.2% (24.4–34.1%)** | **18.5% (14.3–23.0%)** |
| High-grade Serous | No | 944 / 387 | 96.9% (96.1–97.5%) | 79.9% (77.1–82.4%) | 63.0% (58.8–66.9%) | 53.2% (48.3–57.7%) |
|  | Yes | 543 / 407 | 92.0% (90.2–93.5%) | 55.4% (50.4–60.0%) | 29.6% (24.6–34.8%) | 18.9% (14.6–23.6%) |
| Low-grade Serous | No | 87 / 26 | 97.9% (95.0–99.1%) | 86.6% (77.5–92.1%) | 73.8% (61.3–82.8%) | 65.4% (50.3–76.9%) |
|  | Yes | 31 / 16 | 95.9% (90.6–98.2%) | 75.0% (59.5–85.3%) | 54.7% (34.9–70.7%) | * |
| Carcinosarcoma | No | 35 / 17 | 95.5% (92.3–97.4%) | 72.4% (57.5–82.9%) | 51.5% (32.2–67.8%) | 40.3% (21.3–58.7%) |
|  | Yes | 25 / 22 | 85.5% (77.8–90.7%) | 33.1% (17.7–49.3%) | * | * |
| Mucinous | No | 14 / 5 | 95.5% (86.2–98.6%) | 82.1% (54.1–93.9%) | 73.4% (38.0–90.6%) | 70.4% (32.7–89.6%) |
|  | Yes | 14 / 12 | 74.5% (55.5–86.3%) | * | * | * |
| Clear Cell | No | 21 / 16 | 87.0% (77.4–92.7%) | 54.9% (35.9–70.3%) | 39.0% (19.7–57.9%) | 34.3% (14.9–54.8%) |
|  | Yes | 15 / 13 | 82.2% (68.6–90.3%) | 43.0% (21.8–62.7%) | 26.6% (8.8–48.6%) | * |

^1^ Relative survival was predicted up until the last event (death) from flexible parametric models for women with stage III EOC, median age 64 years and fully active ECOG performance status at diagnosis.
*After last event

**Supplemental Table 4.** Predicted^1^ three-year relative survival (RS) with 95% confidence intervals (CI) from flexible parametric models, by diameter of residual disease in the abdomen following cytoreductive surgery of stage III/IV epithelial ovarian cancer (EOC), 2013–2022 (N=501).

| **Residual disease diameter** | **All histotypes**  **501 patients / 358 deaths** | **High-grade serous**  **418 patients / 298 deaths** |
| --- | --- | --- |
|  | **3-year RS (95% CI)** | **3-year RS (95% CI)** |
| 0.2 cm | 57.0% (44.5–67.6%) | 56.5% (46.5–65.3%) |
| 0.5 cm | 48.9% (34.9–61.5%) | 54.7% (44.4–63.8%) |
| 1.0 cm | 46.4% (34.2–57.7%) | 51.2% (42.0–59.6%) |
| 2.0 cm | 46.6% (34.7–57.7%) | 50.6% (42.0–58.6%) |

^1^ Three-year relative survival was predicted from flexible parametric models for women with stage III EOC, median age 64 years and fully active ECOG performance status at diagnosis. Predictions were made from flexible models with the log of residual disease diameter modelled as a restricted cubic spline with two degrees of freedom (figure 2B).

**Supplemental Table 5.** Residual disease diameter and systemic anti-cancer therapy. Surgically treated patients with stage III/IV epithelial ovarian cancer, 2019–2022^1^ (N=725).

|  | **All histotypes, N (%)** | | | **High-grade serous, N (%)** | | |
| --- | --- | --- | --- | --- | --- | --- |
| **Variable** | **Stage III/IV** | **III** | **IV** | **Stage III/IV** | **III** | **IV** |
| **Total** | **725 (100.0)** | **475 (100.0)** | **250 (100.0)** | **616 (100.0)** | **395 (100.0)** | **221 (100.0)** |
| **Diameter of residual disease in abdominal cavity** |  |  |  |  |  |  |
| No residual disease | 522 (75.4) | 356 (77.9) | 166 (70.6) | 442 (75.3) | 294 (77.8) | 148 (70.8) |
| 0.1–0.4 cm | 53 (7.7) | 32 (7.0) | 21 (8.9) | 46 (7.8) | 26 (6.9) | 20 (9.6) |
| 0.5–0.9 cm | 22 (3.2) | 11 (2.4) | 11 (4.7) | 20 (3.4) | 10 (2.6) | 10 (4.8) |
| 1.0 cm | 27 (3.9) | 17 (3.7) | 10 (4.3) | 22 (3.7) | 13 (3.4) | 9 (4.3) |
| 1.1–2.9 cm | 47 (6.8) | 28 (6.1) | 19 (8.1) | 39 (6.6) | 24 (6.3) | 15 (7.2) |
| 3.0–20.0 cm | 21 (3.0) | 13 (2.8) | 8 (3.4) | 18 (3.1) | 11 (2.9) | 7 (3.3) |
| Unknown diameter | 33 | 18 | 15 | 29 | 17 | 12 |
| **Type of chemotherapy** |  |  |  |  |  |  |
| None | 23 (3.2) | 17 (3.6) | 6 (2.4) | 15 (2.4) | 9 (2.3) | 6 (2.7) |
| Neoadjuvant | 24 (3.3) | 15 (3.2) | 9 (3.6) | 22 (3.6) | 14 (3.5) | 8 (3.6) |
| Neoadjuvant and adjuvant | 313 (43.2) | 170 (35.8) | 143 (57.2) | 291 (47.2) | 154 (39.0) | 137 (62.0) |
| Adjuvant after surgery | 365 (50.3) | 273 (57.5) | 92 (36.8) | 288 (46.8) | 218 (55.2) | 70 (31.7) |
| **Neoadjuvant chemotherapy regimen, N=337** |  |  |  |  |  |  |
| Platinum-based + taxane, 1-3 cycles | 125 (37.1) | 74 (40.0) | 51 (33.6) | 116 (37.1) | 67 (39.9) | 49 (33.8) |
| Platinum-based + taxane + bevacizumab, 1-3 cycles | 75 (22.3) | 39 (21.1) | 36 (23.7) | 69 (22.0) | 35 (20.8) | 34 (23.4) |
| Platinum-based + taxane, 4-6 cycles | 84 (24.9) | 46 (24.9) | 38 (25.0) | 80 (25.6) | 43 (25.6) | 37 (25.5) |
| Platinum-based + taxane + bevacizumab, 4-6 cycles | 52 (15.4) | 26 (14.1) | 26 (17.1) | 47 (15.0) | 23 (13.7) | 24 (16.6) |
| Platinum-based + taxane, > 6 cycles | 1 (0.3) | 0 (0.0) | 1 (0.7) | 1 (0.3) | 0 (0.0) | 1 (0.7) |
| **Adjuvant chemotherapy regimen, N=678** |  |  |  |  |  |  |
| Platinum-based + taxane, 1-3 cycles | 135 (19.9) | 76 (17.2) | 59 (25.1) | 119 (20.6) | 64 (17.2) | 55 (26.6) |
| Platinum-based + taxane + bevacizumab, 1-3 cycles | 158 (23.3) | 82 (18.5) | 76 (32.3) | 147 (25.4) | 75 (20.2) | 72 (34.8) |
| Platinum-based + taxane, 4-6 cycles | 225 (33.2) | 196 (44.2) | 29 (12.3) | 178 (30.7) | 158 (42.5) | 20 (9.7) |
| Platinum-based + taxane + bevacizumab, 4-6 cycles | 87 (12.8) | 36 (8.1) | 51 (21.7) | 75 (13.0) | 33 (8.9) | 42 (20.3) |
| Platinum-based + taxane, > 6 cycles | 11 (1.6) | 11 (2.5) | 0 (0.0) | 9 (1.6) | 9 (2.4) | 0 (0.0) |
| Platinum-based + taxane + bevacizumab, > 6 cycles | 3 (0.4) | 2 (0.5) | 1 (0.4) | 2 (0.3) | 1 (0.3) | 1 (0.5) |
| Platinol alone | 50 (7.4) | 34 (7.7) | 16 (6.8) | 45 (7.8) | 30 (8.1) | 15 (7.2) |
| Taxan/other chemotherapy alone | 7 (1.0) | 5 (1.1) | 2 (0.9) | 2 (0.3) | 1 (0.3) | 1 (0.5) |
| PARP-inhibitors alone | 2 (0.3) | 1 (0.2) | 1 (0.4) | 2 (0.3) | 1 (0.3) | 1 (0.5) |
| **Adjuvant bevacizumab or PARP-inhibitor, N=678** |  |  |  |  |  |  |
| Neither | 321 (47.3) | 242 (54.6) | 79 (33.6) | 254 (43.9) | 188 (50.5) | 66 (31.9) |
| Bevacizumab | 195 (28.8) | 95 (21.4) | 100 (42.6) | 169 (29.2) | 83 (22.3) | 86 (41.5) |
| PARP-inhibitor | 94 (13.9) | 69 (15.6) | 25 (10.6) | 90 (15.5) | 66 (17.7) | 24 (11.6) |
| Both | 68 (10.0) | 37 (8.4) | 31 (13.2) | 66 (11.4) | 35 (9.4) | 31 (15.0) |
| **Adjuvant bevacizumab among patients treated with primary surgery, N=365** |  |  |  |  |  |  |
| No | 263 (72.1) | 228 (83.5) | 35 (38.0) | 205 (71.2) | 180 (82.6) | 25 (35.7) |
| Yes | 102 (27.9) | 45 (16.5) | 57 (62.0) | 83 (28.8) | 38 (17.4) | 45 (64.3) |
| **Adjuvant bevacizumab among patients treated with neoadjuvant chemotherapy and interval surgery, N=313** |  |  |  |  |  |  |
| No | 152 (48.6) | 83 (48.8) | 69 (48.3) | 139 (47.8) | 74 (48.1) | 65 (47.4) |
| Yes | 161 (51.4) | 87 (51.2) | 74 (51.7) | 152 (52.2) | 80 (51.9) | 72 (52.6) |

Abbreviations: PARP: Poly ADP Ribose Polymerase.

^1^ Patients with no information on systemic anti-cancer therapy were excluded; N=1044 patients diagnosed before 2019 and N=80 residing in Northern Norway.

**Supplemental Table 6.** Predicted^1^ relative survival (RS) with 95% confidence intervals (CI) from flexible parametric models, by type of surgery and residual disease status. Patients with stage III/IV invasive epithelial ovarian cancer who received adjuvant chemotherapy after cytoreductive surgery, 2019–2022 (N=667).

| **Group** | **Residual disease** | **Patients / deaths** | **1-year RS (95% CI)** | **3-year RS (95% CI)** |
| --- | --- | --- | --- | --- |
| **All histotypes, N=667** |  |  |  |  |
| Primary surgery and adjuvant chemotherapy | No | 266 / 44 | 98.0% (95.2–99.2%) | 87.9% (82.5–91.7%) |
|  | Yes | 94 / 46 | 94.5% (88.9–97.3%) | 53.5% (39.9–65.3%) |
| Neoadjuvant surgery and interval surgery | No | 216 / 61 | 95.7% (92.6–97.6%) | 70.3% (60.4–78.2%) |
|  | Yes | 91 / 51 | 87.3% (79.5–92.3%) | 40.0% (26.8–52.8%) |
| **High-grade Serous, N=571** |  |  |  |  |
| Primary surgery and adjuvant chemotherapy | No | 211 / 33 | 99.0% (96.0–99.8%) | 89.7% (83.8–93.6%) |
|  | Yes | 75 / 35 | 97.3% (91.4–99.1%) | 60.2% (45.6–72.1%) |
| Neoadjuvant surgery and interval surgery | No | 200 / 55 | 97.2% (94.4–98.6%) | 72.9% (62.7–80.8%) |
|  | Yes | 85 / 47 | 90.0% (82.6–94.3%) | 42.8% (28.7–56.2%) |

^1^Relative survival was predicted for patients aged 64 years at diagnosis, stage III and ECOG performance status zero (fully active).
