# Supplementary material for: Ovarian cancer survival by residual disease following cytoreductive surgery: a nationwide study in Norway
Source: Br J Cancer. 2025 Apr 26;132(12):1158–66. doi: 10.1038/s41416-025-03018-0 (PMC12152144; doi:10.1038/s41416-025-03018-0)

Supplemental Figure 1. Kaplan-Meier survival estimates and log-rank test for difference in survival by histotype and residual disease status after cytoreductive surgery. Patients diagnosed with stage III/IV invasive epithelial ovarian cancer during 2013–2022 (N=1797).

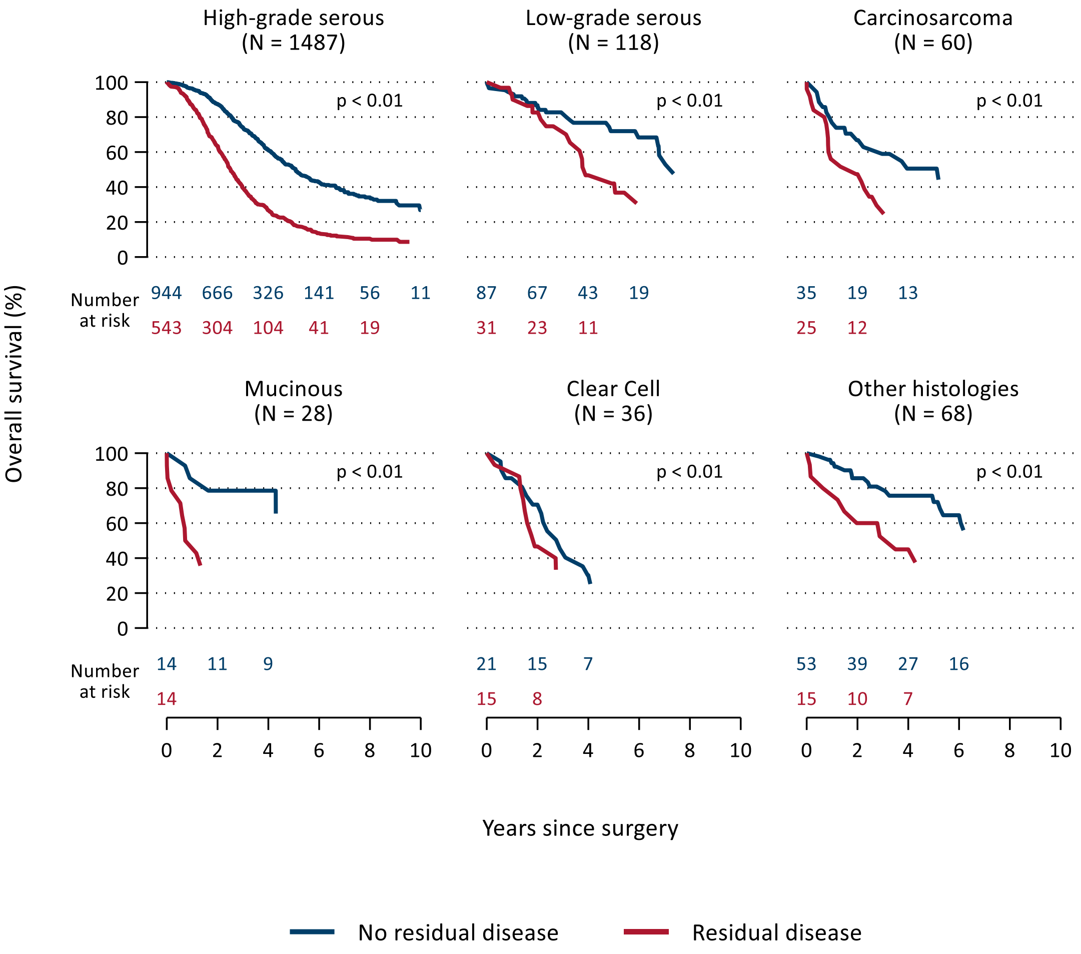

Supplement: Supplementary file 2 — Supplementary Figure 1 [file 41416_2025_3018_MOESM2_ESM.pdf]
